# Isolation, identification and pathogenicity analysis of BCoV epidemic strains in Xinjiang

**DOI:** 10.3389/fmicb.2026.1788466

**Published:** 2026-03-30

**Authors:** Fang Min, Yumeng Liang, Qian Jiang, Xinyu Tao, Jianlong Wan, Tingting Xue, Na Li, Rulong Chen, Qi Zhong, Gang Yao, Xuelian Ma

**Affiliations:** 1 College of Veterinary Medicine, Xinjiang Agricultural University, Urumqi, China; 2 Xinjiang Key Laboratory of New Drug Research and Development for Herbivores, Urumqi, China; 3 Institute of Veterinary Research, Xinjiang Academy of Animal Science, Urumqi, China

**Keywords:** bovine coronavirus, genomic analysis, pathogenicity, virus isolation, virus purification

## Abstract

Bovine coronavirus (BCoV) causes diarrhea in calves, winter dysentery in adult cattle, and respiratory diseases, posing a significant threat to the cattle industry. In this study, a BCoV strain was isolated from intestinal lymph node tissues of infected calves in Hami, Xinjiang, using HRT-18G cells with an optimized trypsin-HEPES synergistic culture system. Following plaque purification, the virus was confirmed by RT-PCR, indirect immunofluorescence assay, and transmission electron microscopy, and designated as BCoV-XJHM. The viral titer reached 10^8.0^ TCID_50_/mL. Whole-genome sequencing revealed that BCoV-XJHM shares 98.4–99.2% nucleotide identity with 35 representative domestic strains, clustering with the Guangxi strain (GX-NN230328, PP599028.1) in the same evolutionary subclade. Two amino acid substitutions (S81A and S149A) were observed in the N-terminal domain of the nucleocapsid (N) protein. In a BALB/c mouse model, oral inoculation of BCoV-XJHM induced significant body weight loss (*P* < 0.001) and mild pulmonary pathology, with viral RNA detected in lung and colon tissues. This study reports an optimized protocol for BCoV isolation in HRT-18G cells, describes two amino acid substitutions in the N protein of the BCoV-XJHM strain, and establishes a BALB/c mouse model for evaluating BCoV pathogenicity in a heterologous host.

## 1 Introduction

Bovine coronavirus (BCoV) is one of the main pathogens that causes diarrhea in calves and can infect both the digestive tract and respiratory tract (Geng et al., 2023). The disease has a rapid onset and a long course, which is also an important factor leading to the high incidence of diarrhea in calves in Xinjiang (Pratelli et al., 2021). Since bovine coronavirus (BCoV) was first isolated in the United States in 1972 (Charles et al., 1973), it has been recognized as a significant pathogen in cattle populations worldwide. In China, serological surveys since the 1990s have confirmed its widespread prevalence, with antibody positivity rates ranging from 38.89 to 86.4% across multiple provinces (Jiang et al., 2007; Liu et al., 2009; Fu et al., 2012), with a national fecal detection rate of 32.12% (Li, 2024). Recent regional studies further elucidate this distribution. Antigen positivity rates of 23.81% (locally reaching 66.67%) have been reported in Inner Mongolia (Chen et al., 2025). In Heilongjiang Province, BCoV detection rates among diarrhoeic calves were 33.85% (Chen, 2025) and 21.53% (Zhu et al., 2022). In Xinjiang, recorded detection rates were 19.91% (Jia et al., 2024) and 23.93% (Wang et al., 2023). A Sichuan study reported a detection rate of 16.8% in diarrhoeic cattle (Deng et al., 2021), while a survey of affected calves across 14 provinces revealed a BCoV detection rate of 21.59% (Yang et al., 2019). Collectively, these data confirm the persistent circulation of BCoV within Chinese cattle populations. Beyond cattle, BCoV has been detected in wild ruminants including camelids and elk (Huang et al., 2023), indicating broader host range. Infected animals shed the virus via feces, with viral shedding persisting for up to 18 months post-recovery (Kanno et al., 2018), resulting in significant economic losses to the livestock industry (Madin et al., 1956; Collins et al., 1987; Ingrid et al., 2017; Yan et al., 2025).

BCoV belongs to the *Betacoronavirus* genus of the *Coronaviridae* family. It is a single-stranded straight-stranded RNA virus with a capsule. Its morphology is spherical, and the approximate diameter ranges from 60 to 220 nm. It is named because its virus particles show a crown-like structure when observed under an electron microscope (Aspen et al., 2022). Among RNA viruses, BCoV has the largest genome, with lengths of approximately 26 and 32 kb. The two ends of the BCoV genome contain 5’UTR and 3’UTR nontranslated regions, and the middle part contains 10 open reading frames. These open reading frames encode 5 structural proteins, including hemagglutinin-esterase glycoprotein (HE), spike (S), envelope (E), membrane glycoprotein (M) and nucleocapsid (N) proteins. The remaining open reading frames encode nonstructural proteins. Due to the high similarity of the N protein between different BCoV strains, it is often used for diagnosis (Cyr-Coats et al., 1988; Su-Jin et al., 2006; Dinesh, 2020; Wen, 2023).

To date, the separation of BCoV still faces great challenges. The main reason is that its capsule is easily damaged. During the freeze—thaw process, the spike (S) protein easily falls off, resulting in the inactivation of the virus and difficulty in obtaining complete infectious particles (Liu et al., 2023). Therefore, in *in vitro* culture, trypsin must be added to produce S1/S2 subfragments through the enzymatic cleavage of the S protein to mediate viral invasion of host cells (Elisabete et al., 2008; Hodnik et al., 2020). Although some progress has been achieved in determining the pathogenesis, epidemic characteristics and evolutionary laws of BCoV, information on the stable passage of strains is still lacking (Liu, 2013; Lu et al., 2018; Qiang et al., 2018; Giovanni et al., 2020; Anastasia and Linda, 2021). In addition, the genomic characteristics of BCoV and its pathogenic mechanism have not been systematically clarified. Research on the genetic diversity and adaptive evolution of epidemic strains is still insufficient, especially in main livestock-producing areas such as Xinjiang (Wang et al., 2023).

In this study, a strain of BCoV was successfully isolated from the lymph nodes of infected calf intestines in a BCoV-infected cattle farm in Hami, Xinjiang. Based on the latest methods released by the BCoV Classification Working Group, we identified this strong strain, analyzed the genetic evolution of its genome, and evaluated its pathogenicity in mice. This study aims to elucidate the evolution of BCoV and the host interaction mechanism and provide a scientific basis for promoting targeted prevention and control strategies and the research and development of BCoV vaccines.

## 2 Materials and methods

### 2.1 Clinical samples, cell lines, and experimental animals

The intestinal lymph node tissues was collected from infected calves in a BCoV-infected cattle farm in Hami, Xinjiang, and stored at –80°C for later use. Human rectal tumor-18G (HRT-18G) cells are preserved in our laboratory. Four-week-old SPF BALB/c mice are purchased from the Experimental Animal Center of Houbo College of Xinjiang Medical University, Xinjiang Uygur Autonomous Region.

### 2.2 Main reagents

TRIzol reagent (Invitrogen, Carlsbad, United States). Hifair^®^ first-strand cDNA synthesis kit, TransScript one-step gDNA removal and cDNA synthesis premix, 2 × Taq PCR Master Mix, and DL2000 DNA Marker (Yisheng Biotechnology Company, Shanghai, China). RPMI 1640 medium and fetal bovine serum (Biological Industries (BI), Beit Haemek, Israel). 1 M HEPES, penicillin–streptomycin dual antibiotics and cell cryopreservation solution (Beijing Soleibao Technology Co., Ltd., Beijing, China). Trypsin solution B (Shanghai Dateer Co., Ltd., Shanghai, China). FITC-labeled rabbit anti-bovine IgG and DAPI staining solution (Abcam, Cambridge, UK). BCoV-positive bovine serum was collected and stored in our laboratory.

### 2.3 Nucleic acid extraction and virus detection

Total RNA was extracted from each sample using TRIzol reagent, and cDNA was synthesized using the Yisheng Hifair^®^ III First-Strand cDNA Synthesis Kit according to the manufacturer’s instructions. Reverse transcription PCR (RT-PCR) was performed to identify bovine coronavirus (BCoV), bovine viral diarrhea virus (BVDV), bovine nebovirus (BNeV), bovine norovirus (BNoV), bovine rotavirus (BRV) and bovine parvovirus (BPV) in the samples. The reaction system (15 μL) contained 7.5 μL of 2 × Es Taq PCR Master Mix, 0.5 μL of upstream and downstream primers, and 4.5 μL of RNase-free ddH_2_O. The thermal cycling procedure was as follows: 95°C predenaturation for 5 min, 95°C denaturation for 30 s, annealing for 30 s (see Table 1 for the annealing temperature), and extension at 72°C for 30 s for a total of 35 cycles, followed by a final extension at 72°C for 10 min. After the reaction, agarose gel electrophoresis and sequencing were performed on the RT—PCR products (Table 1).

**TABLE 1:** Information on the primer and probe sequences.

Virus	Primer sequences (5’→3’)	Target gene	NCBI registration number	Annealing temperature
BCoV	F:TGTACCCTACTATTCTTGGTTCTCTG	N	MW711287.1	55°C
	R:TCTGCTTAGTTACTTGCTGTGGC			
BCoV-QP	F:GATCTACTTCACGCGCATCC	N		60°C
	R:GTGGCTTAGTGGCATCCTTG			
BCoV-QP	F1:FAM-TGGCTCTACTGCGCGATCCTGCA-BHQ1	N		60°C
BVDV	F:AGTCGTCAGTGGTTCGAC	5’UTR	KF501393	47 °C
	R:TCCATGTGCCATGTACA			
BNeV	F:GTGTCGGGYCCWGTGTTCCT	RdRp	MK620281.1	55°C
	R:AAATAGCACGGGCTTCTTC			
BNoV	F:ATCTCGCACGATGCCAAG	RdRp	MN122335	55°C
	R:GTCATCTTCATTTACAAAATCGG			
BRV	F:GTACGATGTGGCTAAACGC	VP6	OR205420.1	55°C
	R:ATGCTGCTACTGCTGGTGT			
BPV	F:GACCATTTTTAGCGAAAACATTGT	VP2	MW032436.1	55°C
	R:GTTGTTGTCTACGACTGAGATTGGA			

In addition, reverse transcription quantitative PCR (RT-qPCR) was used to identify bovine coronavirus (BCoV) in each generation. The reaction system (20 μL) contained 10 μL of reaction premix, 7.8 μL of DEPC-treated water, 0.4 μL of upstream and downstream primers (10 μM), 0.4 μL of probe (10 μM), and 1.0 μL of DNA template. The amplification conditions included predenaturation at 94°C for 30 s, denaturation at 94°C for 5 s, and 40 cycles of amplification 60°C for 30 s, and fluorescence collection at 60°C (Table 1).

### 2.4 Virus isolation and plaque purification

Intestinal lymph node tissues confirmed BCoV-positive by RT—PCR were homogenized, filtered, and inoculated onto HRT-18G cell monolayers at approximately 90% confluence. To optimize culture conditions, we tested HEPES at 50/100/150 mM (with 12.5 μg/mL trypsin B for 10/20/30 min) during cell pretreatment, and at 30/50/70/100 mM in maintenance medium. Briefly, cells were washed twice with PBS (1% P/S), stimulated with trypsin/HEPES medium for the specified time, and inoculated with viral solution for 4 h at 4°C. After rinsing, maintenance medium (with gradient HEPES) was added, and cells were incubated at 37°C until cytopathic effect (CPE) > 80% (up to 5 d), then harvested and stored at –80°C. For plaque purification, virus was 10-fold serially diluted, inoculated onto cells, and overlaid with agarose-containing medium. After 4–5 days, single plaques were picked, amplified, and the process repeated twice to obtain a purified virus.

### 2.5 Identification of isolated strains

#### 2.5.1 RT-PCR identification

The cell culture of the isolate was used, total RNA was extracted, cDNA was reverse transcribed from RNA, the N gene-specific primer described in section 2.3 was used for PCR amplification, and the amplification product was subjected to agarose gel electrophoresis and send to Shanghai Shenggong Bioengineering Co., Ltd., for sequencing and identification. The sequencing results were compared, analyzed and verified on the NCBI website.

#### 2.5.2 Identification of immunofluorescence assay

The isolated strains were inoculated in HRT-18G cells. When the CPE appeared, the cells were fixed with 4% polyformaldehyde, permeabilized with 0.5% Triton X-100, and blocked with 1% BSA. After sequential incubations with the bovine anti-BCoV-positive serum (1:200) and FITC-labeled rabbit anti-cow IgG (1:500), the nuclei were stained with DAPI, the excess DAPI was removed with PBS, and the cells were observed with a laser confocal microscope.

#### 2.5.3 Transmission electron microscope observation

The virus-containing supernatant was filtered through a 0.45 μm filter membrane by centrifugation at 10,000 × g centrifuge for 10 min and then collected and sent to the Wuhan Sevier Experimental Center to observe the morphology of the virus particles using a transmission electron microscope.

### 2.6 Determination of median tissue culture infectious dose (TCID_50_)

The isolated and purified virus was diluted 10 times, HRT-18G cells were inoculated with the virus in a 96-well plate, with 8 wells established per dilution. At the same time, a normal cell control was established. The CPE was observed daily, and the Reed–Muench method was used to calculate the TCID_50_ (Yuchen et al., 2023).

### 2.7 Genetic evolutionary analysis of isolates

The nucleic acids of the isolated Xinjiang BCoV strain were sent to Shanghai Tanpu Biotechnology Co., Ltd., for sequencing, and the obtained sequences were analyzed, assembled, spliced and calibrated using SPAdes, MEGAHIT and SeqMan software. The Cluster W algorithm in MEGA software was used to compare the sequence of the isolated strain with the BCoV reference sequence published in GenBank, and 35 representative BCoV strains in the NCBI GenBank database were selected.

Sequence similarity was initially evaluated using Basic Local Alignment Search Tool nucleotide (BLASTn) for preliminary assessment. Subsequently, 35 representative strains were selected and subjected to whole-genome multiple sequence alignment employing the ClustalW algorithm implemented in MEGA 12.0 software. Based on this alignment, nucleotide identity among the strains was further precisely calculated using the MegAlign program (DNAstar), and a phylogenetic tree was constructed based on the maximum likelihood (ML) method with the Kimura two-parameter (K2) model and 1,000 bootstrap replicates. Additionally, to visualize mutation sites, 20 sequences belonging to the same evolutionary clade as the strain studied here were selected, and amino acid sequence alignment of hypervariable regions in the structural proteins (HE, S, E, M, N) was performed using ESPript 3.0.

### 2.8 Pathogenicity analysis of isolates

Four-week-old BALB/c mice were randomly divided into the infected group and the control group (12 mice in each group), for a total of 24 mice. The inoculation dose was 1 × 10^8^ TCID_50_ per mouse, delivered by oral gavage. Control animals were inoculated with an equal volume of cell culture medium.

After the viral infection, vital signs and clinical manifestations were observed daily. Body temperature was monitored using a digital rectal thermometer at a fixed time each day, with three consecutive measurements averaged for each recording. The normal core body temperature range for mice (35.2–37.8°C) served as the physiological reference (Jordan, 1995; National Research Council, 2006; Gordon, 2017; Hankenson et al., 2018). On days 1, 5, and 10 post-infection, four mice from each group were euthanized by cervical dislocation. Death was then confirmed. Both lung and colon tissues were collected. For each tissue type, one sample was used for RT-PCR detection of the virus, and the other was fixed with 10% formaldehyde, embedded in paraffin, sectioned, and stained with HE to observe pathological changes in the tissue.

Body weight and body temperature data were analyzed using two-way repeated measures ANOVA, with group and time as factors. *Post hoc* comparisons were performed using Tukey’s test. Statistical significance was set at *P* < 0.05. All analyses were conducted using GraphPad Prism (version 9.0).

## 3 Results

### 3.1 RT—PCR identification of disease materials

**FIGURE 1:**
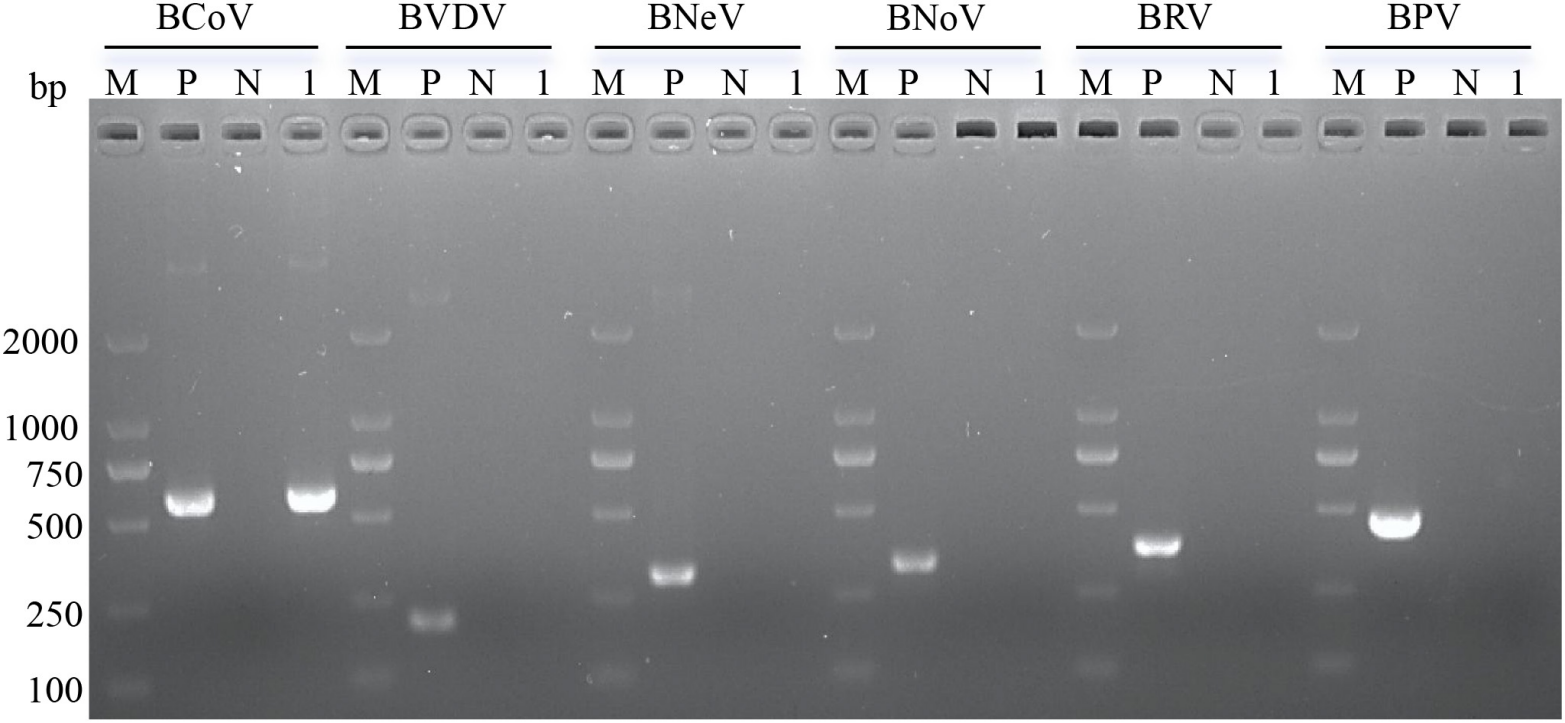
RT—PCR results for the samples. M, DL 2000 DNA Marker; P, positive control; N, negative control; 1, sample.

The results of 1.5% agarose gel electrophoresis revealed a band at 596 bp following the electrophoresis of the amplification product obtained using the BCoV-N-F/R primers. Sequencing confirmed that this fragment matched the BCoV-N primer amplification product, with no evidence of BVDV, BNeV, BNoV, BRV, or BPV infection (Figure 1).

### 3.2 Virus isolation and culture

**FIGURE 2:**
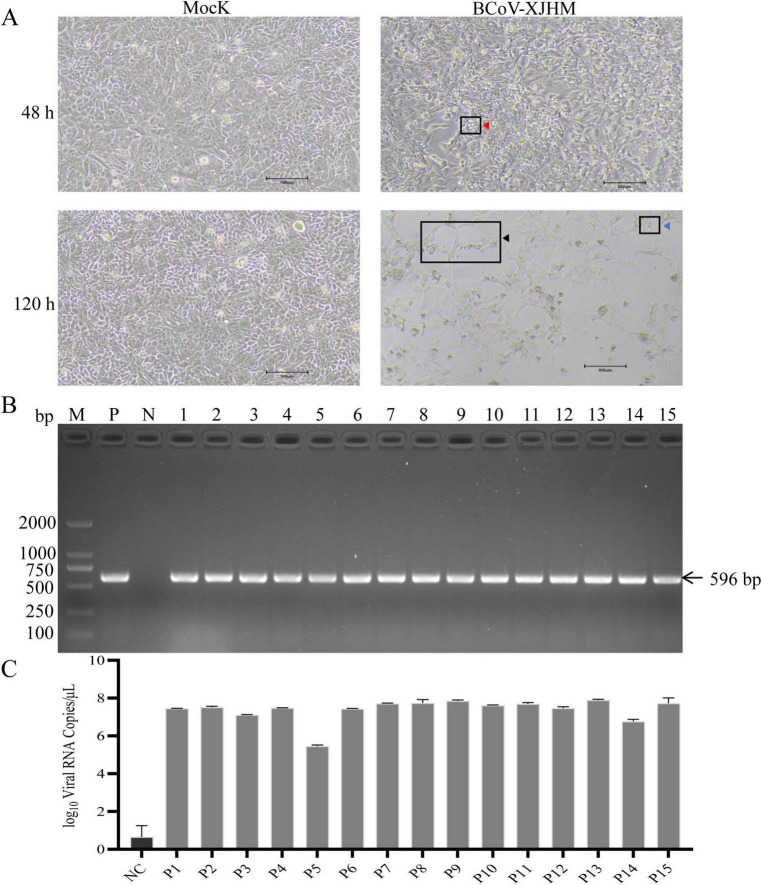
Virus isolation and identification. **(A)** Cell pathology results. **(B)** Diagram showing of the transmission of 15 generations of the virus in the cell culture supernatant based on the RT—PCR results. M, DL 2000 DNA Marker; P, positive control; N, negative control; 1–15, supernatants from the blind transmission of 1st–15th-generation viruses. **(C)** Fluorescence quantitative PCR was used to detect the viral RNA copy numbers in different samples (P1–P15).

After filtration, BCoV-containing supernatant was inoculated onto HRT-18G cells. The optimal pretreatment conditions were 100 mM HEPES with 12.5 μg/mL trypsin B for 20 min, maintaining stable cell density (Supplementary Figure 1A). In maintenance medium, 50 mM HEPES induced the most significant CPE (Supplementary Figure 1B). The cells showed cavities of different sizes (red triangles) at 48 h after viral inoculation, and the cells were rounded, stretched (black triangles), and formed floating bodies (blue triangles) after 120 h (Figure 2A). After 15 consecutive stable passages, RT—PCR products from the culture supernatant consistently showed an expected target band of approximately 596 bp after electrophoresis. Sequencing confirmed that this product was the BCoV-N gene (Figure 2B). These findings were supported by the qPCR results. After 15 consecutive passages, viral copy numbers reached 2.452 × 108 copies/μL, confirming that BCoV can replicate extensively within HRT-18G cells following infection via the passage method established in this study (Figure 2C).

### 3.3 Plaque purification of the isolated strain

**FIGURE 3:**
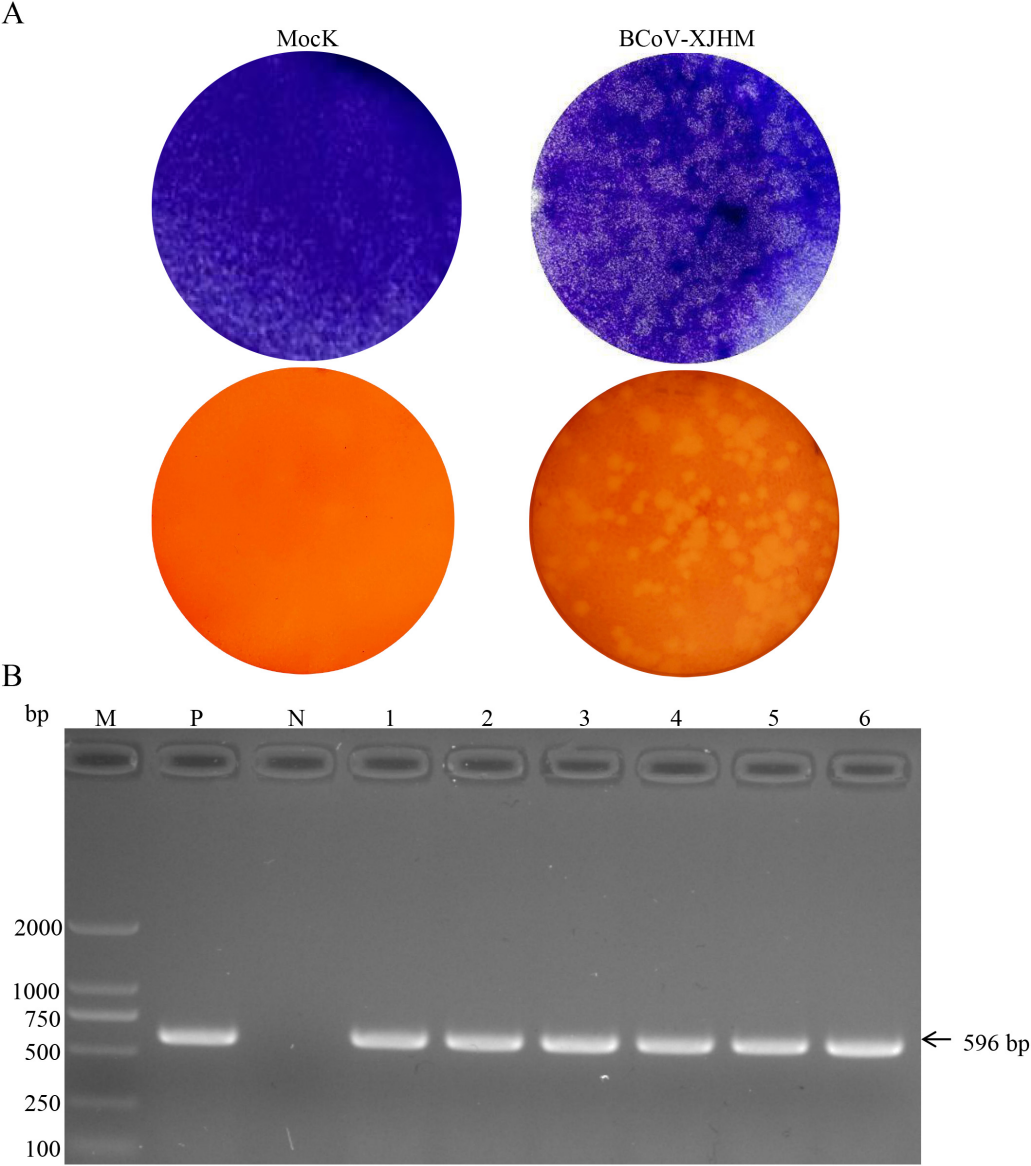
Results of the purification and identification of isolated plaques. **(A)** Diagram showing the purification of plaques of the BCoV isolate. **(B)** RT—PCR analysis of BCoV plaque purification. M, DL 2000 DNA Marker; P, positive control; N, negative control; 1–3, supernatants from first- to third-generation viruses; 4–6, supernatants from second-generation to third-generation viruses.

After two rounds of plaque purification, a monoclonal BCoV strain was successfully obtained (Figure 3A). All subsequent viral passages were confirmed BCoV-positive by RT-PCR, and sequencing verified the amplified products as the BCoV-N gene (Figure 3B).

### 3.4 Identification of isolates and determination of viral titer

**FIGURE 4:**
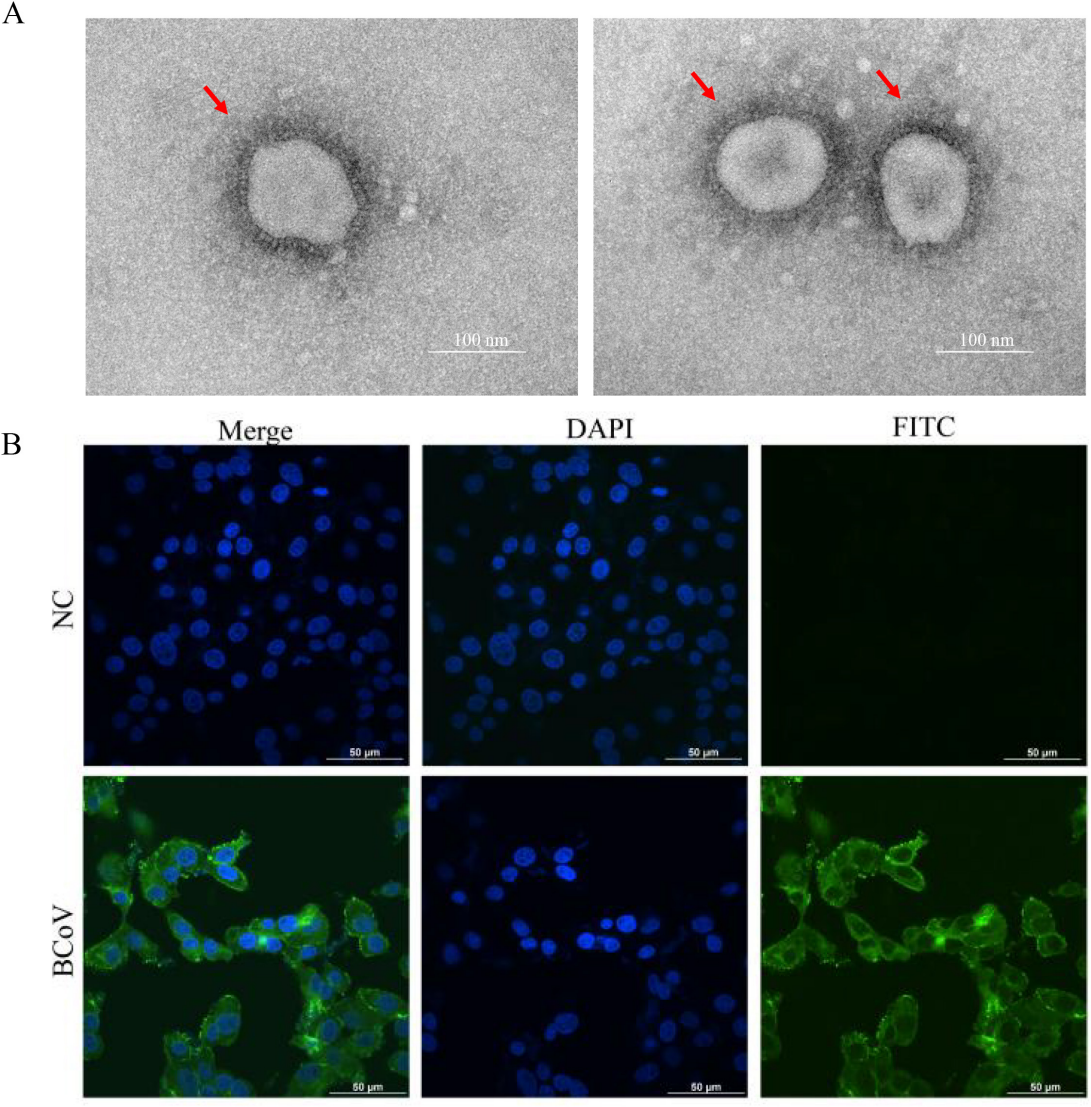
Results for the identification of isolated strains. **(A)** Electron microscopy image of virus particles of the BCoV isolate (the arrow indicates virus particles). **(B)** Indirect immunofluorescence staining of HRT-18G cells after infection (green: viral antigen; blue: DAPI staining).

TEM showed spherical virus particles with a diameter of approximately 80–200 nm and typical coronal protrusions (Figure 4A). IFA showed that specific green fluorescence appeared in the cytoplasm of infected cells, and no fluorescence signal was detected in the negative control (Figure 4B). The BCoV TCID_50_ was calculated using the Reed—Muench formula as 10^8.0^/mL, and the isolate was named BCoV-XJHM.

### 3.5 Genetic evolution analysis of isolates

**FIGURE 5:**
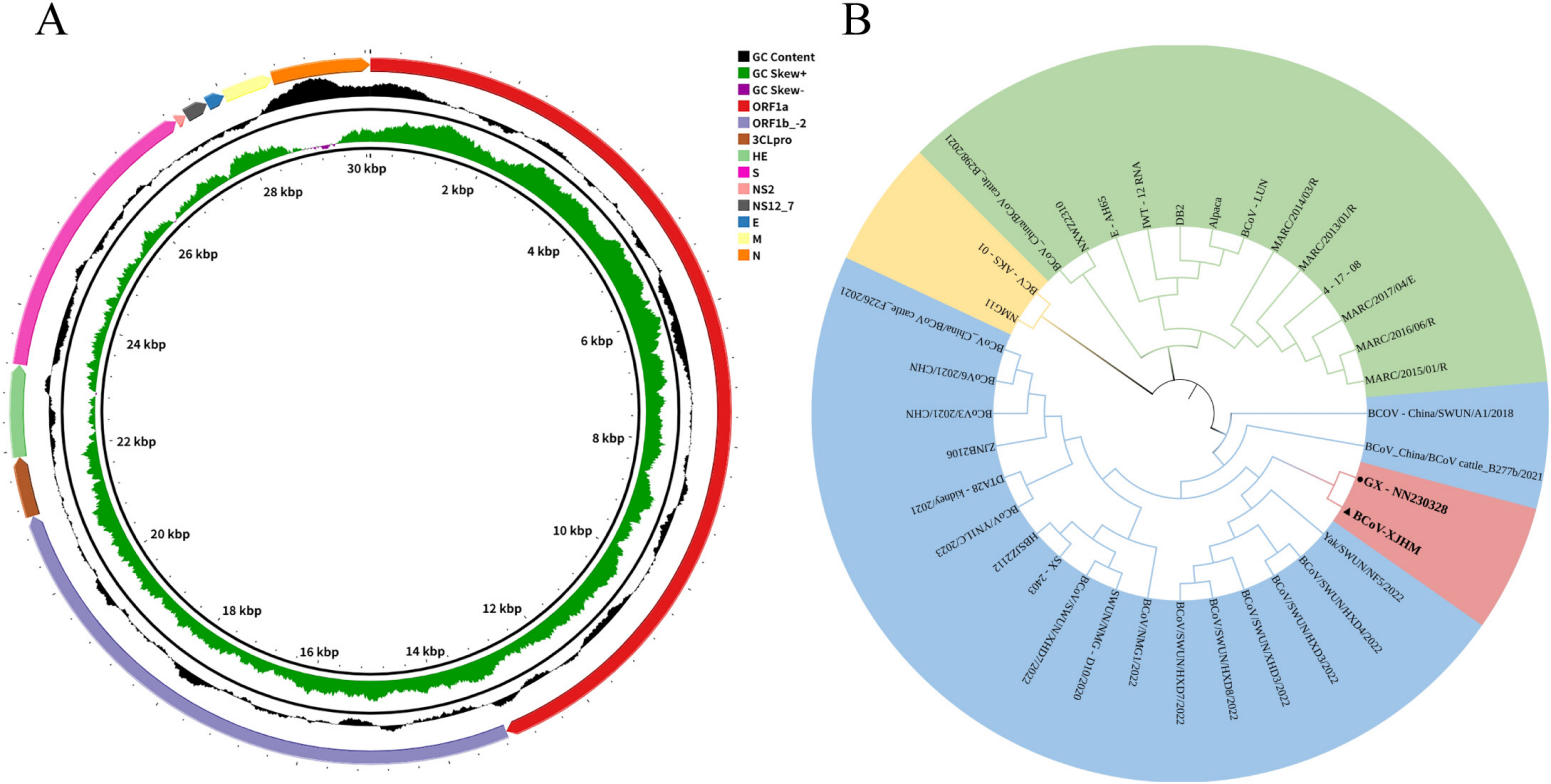
Whole-genome features of the BCoV-XJHM strain. **(A)** Genome structure, GC content distribution, and CDS localization map of BCoV XJHM strain **(B)** Map showing the genetic evolution of the whole-genome nucleotide sequence of the BCoV-XJHM strain. ▲ represents the isolated strain BCoV-XJHM; ● represents the most closely related strain.

Whole-genome sequencing of the BCoV-XJHM isolate was performed using the Illumina 2,500 platform, and a complete genome of 31,053 bp was assembled. The annotated genomic of BCoV-XJHM showing the positions of all coding sequences (CDS) for viral structural and non-structural proteins (e.g., S, E, M, N, and replicase polyproteins) along with the GC content distribution across the genome (Figure 5A). Initial BLASTn analysis identified the Hohhot strain (BCoV/NMG1/2022, OP924545.1) as the closest match (99.41% similarity). Further multiple sequence alignment of 35 representative strains using MegAlign revealed that BCoV-XJHM shared 98.4–99.2% nucleotide identity with them, with the highest identity (99.2%) observed with a group of strains from five Chinese regions, including the BCoV/NMG1/2022 strain (Table 2 and Supplementary Figure 2). A phylogenetic analysis of the whole-genome nucleotide sequence of BCoV XJHM conducted using MEGA 12.0 revealed that BCoV-XJHM clusters with the GX-NN230328 strain into a monophyletic clade. This clustering is supported by a bootstrap value > 70%, indicating high reliability of the phylogenetic relationship (Figure 5B).

**TABLE 2:** Comparison of sequence similarity and homology between the BCoV-XJHM strain and 35 BCoV strains.

GenBank ID	Strain	Similarity (%)	Genome-wide homology analysis (%)	Source
OP924545.1	BCoV/NMG1/2022	99.41	99.2	China—Hohhot
ON142319.1	BCoV6/2021/CHN	99.4	99.2	China—Heilongjiang
OR621174.1	BCoV/SWUN/XHD7/2022	99.36	99.1	China—Heilongjiang
OP866727.1	BCoV_China/BCoV cattle_F226/2021	99.35	99.2	China—Yangling
ON142316.1	BCoV3/2021/CHN	99.33	99.2	China—Taian
OR077317.1	ZJNB2106	99.33	99.0	China—Chengdu
OR077314.1	HBSJZ2112	99.31	99.1	China—Jiangsu
OR612027.1	BCoV/SWUN/HXD7/2022	99.31	99.2	China—Chengdu
OR621175.1	BCoV/SWUN/HXD8/2022	99.31	99.0	China—Sichuan
OP866726.1	BCoV_China/BCoV cattle_B277b/2021	99.3	99.1	China—Guangdong
OR621177.1	BCoV/SWUN/XHD3/2022	99.3	99.1	China—Xinjiang
OR612024.1	BCoV/SWUN/HXD3/2022	99.3	99.1	China—Kunming
OR612025.1	BCoV/SWUN/HXD4/2022	99.3	99.1	United States—Rockville
OR603987.1	Yak/SWUN/NF5/2022	99.27	99.1	China—Sichuan
MW711287.1	SWUN/NMG-D10/2020	99.27	99.1	China—Sichuan
ON544072.1	DTA28-kidney/2021	99.26	99.1	China—Shandong
PQ588962.1	SX-2403	99.25	99.1	China—Inner Mongolia Autonomous Region
MN982198.1	BCOV-China/SWUN/A1/2018	99.19	99.0	China—Sichuan
PQ313104.1	NXWZ2310	99.02	98.8	China—Nanjing
OR088593.1	BCoV/YN1LC/2023	99	98.8	China—Yunnan
KU886219.1	BCV-AKS-01	98.89	98.6	China—Xinjiang
PP352170.1	NMG11	98.88	98.6	China—Inner Mongolia Autonomous Region
PP599028.1	GX-NN230328	98.82	98.6	China—Guangxi
EF424615.1	E-AH65	98.8	98.6	United States—MD
AF391542.1	BCoV-LUN	98.76	98.6	United States—LA
OP866728.1	BCoV_China/BCoV cattle_B298/2021	98.72	98.6	China—Shaanxi
LC494138.1	IWT-12 RNA	98.69	98.4	Japan—Ibaraki
DQ811784.2	DB2	98.66	98.5	United States—MD
MH043954.1	4-17-08	98.66	98.5	United States—PA
DQ915164.2	Alpaca	98.65	98.6	United States—OR
OP037365.1	MARC/2013/01/R	98.64	98.5	United States—NE
OP037371.1	MARC/2015/01/R	98.61	98.4	United States—NE
OP037378.1	MARC/2016/06/R	98.59	98.4	United States—NE
OP037383.1	MARC/2017/04/E	98.59	98.4	United States—NE
OP037369.1	MARC/2014/03/R	98.59	98.4	United States—NE

### 3.6 Analysis of amino acid sequence variation characteristics in BCoV-XJHM isolates

**FIGURE 6:**
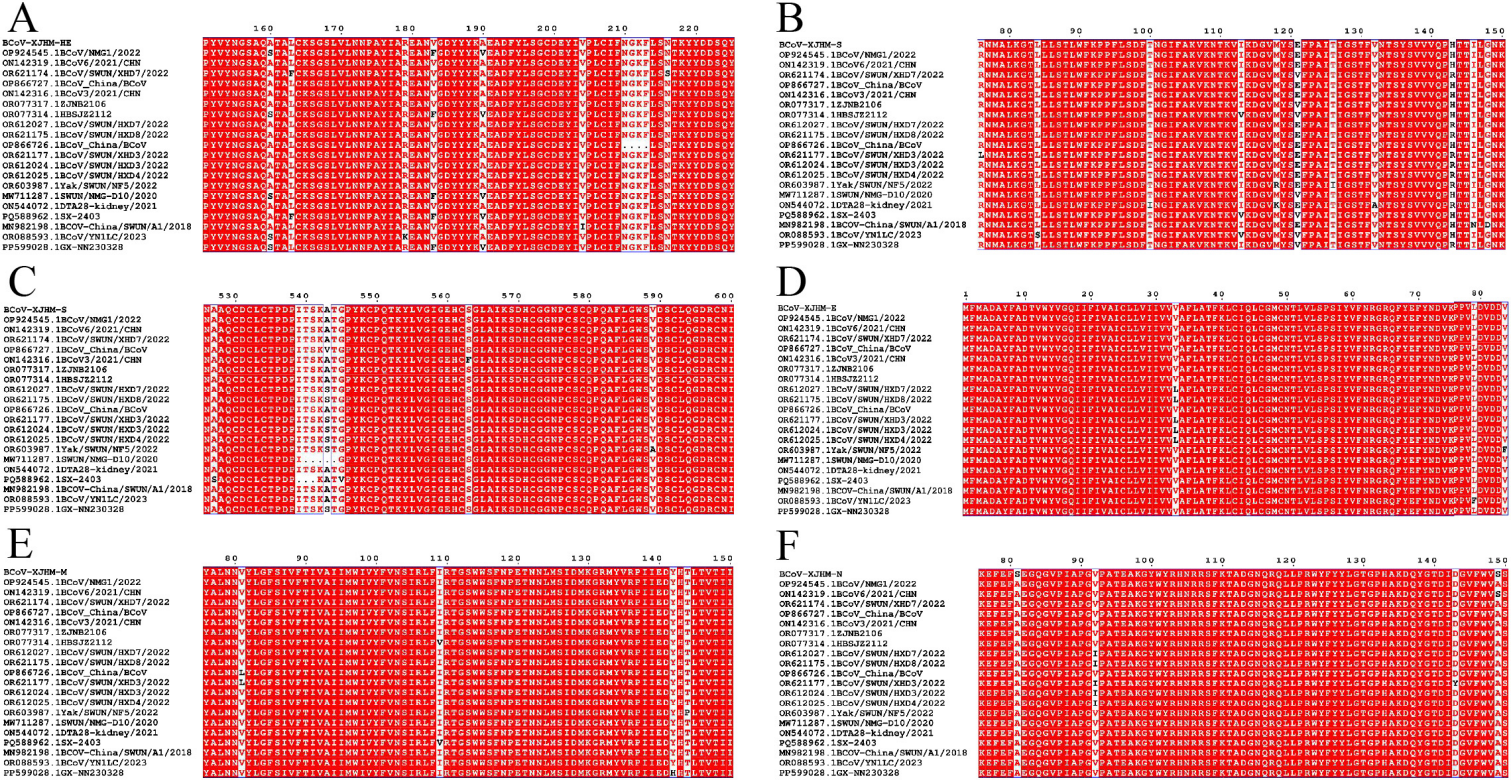
Analysis of amino acid sequence mutations between the BCoV-XJHM strain and the reference strain. **(A)** Amino acid sequence alignment of the BCoV-XJHM-HE protein hypervariable region. **(B,C)** Amino acid sequence alignment of the BCoV-XJHM-S protein hypervariable region. **(D)** Amino acid sequence alignment of the BCoV-XJHM-E protein hypervariable region. **(E)** Amino acid sequence alignment of the BCoV-XJHM-M protein hypervariable region. **(F)** Amino acid sequence alignment of the BCoV-XJHM-N protein hypervariable region. When the similarity score of a column exceeded the threshold, the residue is displayed in red and boxed in blue. Columns with scores below the threshold are displayed in black. Regardless of the threshold value, residues with strict identity are always displayed in white on a red background. Acceptable values were 0–1; the default value of the threshold was 0.7.

Based on the analysis of the evolutionary tree, a mutation analysis was the same branch conducted on 21 strains, including the BCoV-XJHM strain, and the results showed varying degrees of variation in key protein regions (Supplementary Table 1). Mutations of the HE protein at the A160S, V183F, and A190V sites were detected in five strains, while the mutations at other sites were relatively scattered (Figure 6A). The mutation of the S protein was concentrated mainly at three sites: in amino acids 76–148, the H143R mutation was present in 9 strains, followed by E121V. Among amino acids 527–589, the A543V/S mutation was present in 8 strains (Figures 6B,C). V33L mutations in the E protein were relatively common (Figure 6D). M protein mutations were limited and scattered (Figure 6E). Notably, the BCoV-XJHM strain investigated in this study did not contain mutations at the aforementioned sites of the HE, S, E, and M structural proteins. In the relatively conserved BCoV-XJHM-N protein, S81A and S149A mutations were observed (Figure 6F).

### 3.7 Evaluation of pathogenicity in mice

**FIGURE 7:**
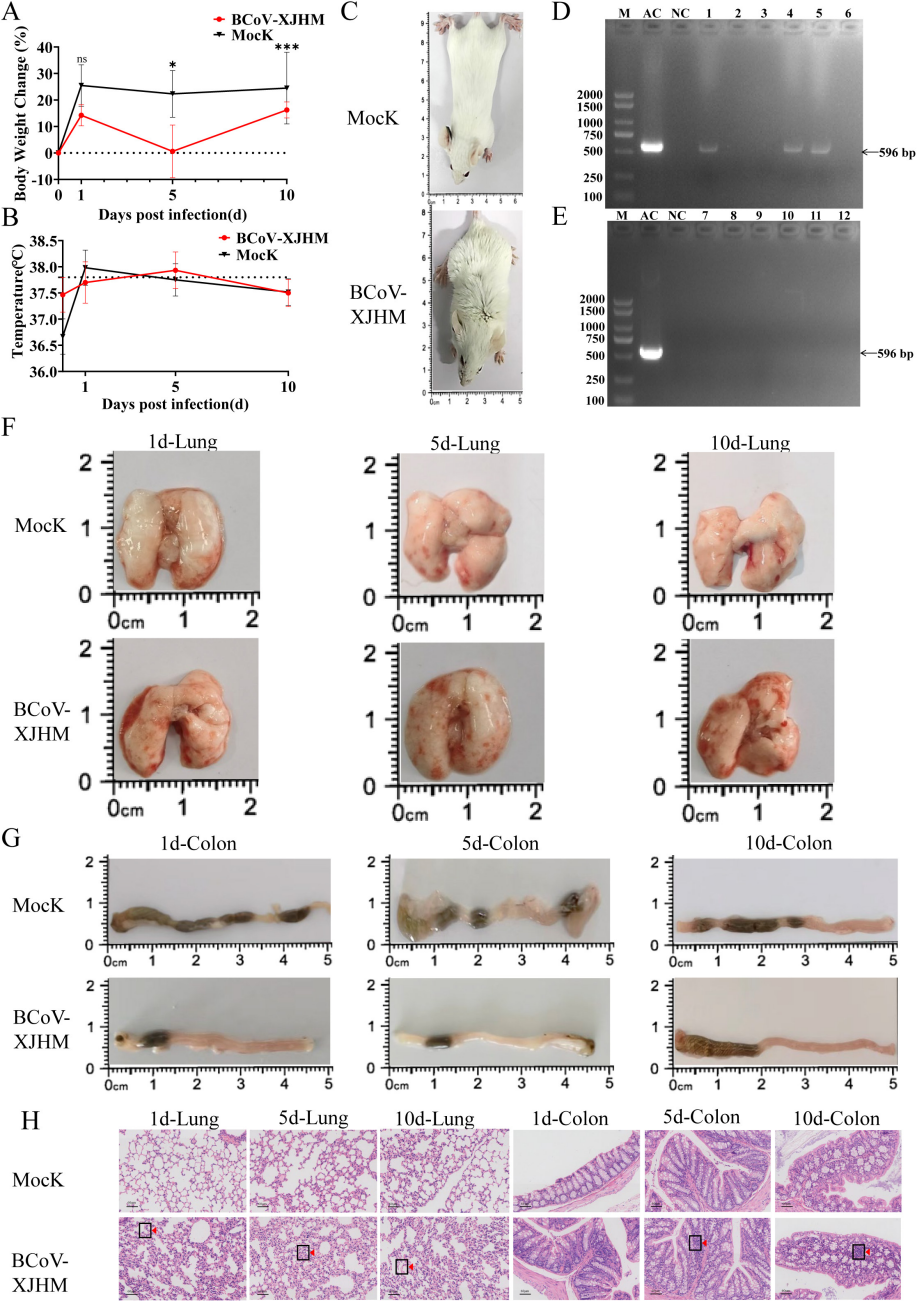
Pathogenicity of BCoV-XJHM in mice (HE, × 20). **(A)** Body weight change of mice following infection. Data are presented as mean ± SEM. Significant differences versus the Mock group at each time point are indicated: *P < 0.05, ** *P* < 0.01, *** *P* < 0.001. The dashed line indicates the baseline (0% change). **(B)** Changes in temperature at 1, 5, and 10 days after infection. **(C)** Phenotypes of the mice. **(D)** RT—PCR detection of lung and colon tissues from the experimental groups at 1, 5, and 10 days after infection. M, DL2000 marker; PV, positive control; NC, negative control; 1–3, lung tissues from the experimental groups at 1, 5, and 10 days after infection; 4–6, colon tissues from the experimental groups at 1, 5, and 10 days after infection. **(E)** RT—PCR detection of lung and colon tissues from the control group at 1, 5, and 10 days after the mock infection. M, DL700 marker; PV, positive control; NC, negative control; 7–9, lung tissues from the control group at 1, 5, and 10 days; 10–12, colon tissues from the control group at 1, 5, and 10 days. **(F)** Observation of lung tissues collected from each group on days 1, 5, and 10 after infection. **(G)** Observation of colon tissues collected from each group on days 1, 5, and 10 after virus infection. **(H)** Images of H&E-stained lung and colon tissues from each group on days 1, 5, and 10 after virus infection, with red arrows indicating inflammatory cell infiltration. The dashed area in **(B)** represents the normal murine core body temperature range (35.2–37.8°C).

BCoV-XJHM-infected mice exhibited typical clinical signs including depression, piloerection, and reduced activity (Figure 7C). Significant body weight suppression was observed in the infected group on day 5 post-infection (p.i.) (*P* < 0.05), which progressed to pronounced weight loss by day 10 (*P* < 0.001) (Figure 7A). Approximately 50% of infected mice showed clinically meaningful weight reduction. Core body temperature remained within the normal range throughout the experiment, with no significant difference between groups (*P* > 0.05) (Figure 7B). BCoV-specific RNA was detected by RT-PCR in lung and colon tissues on the first day after infection, and in colon tissues on day 5 p.i. (Figure 7D). All mock-infected controls were negative (Figure 7E). Autopsy revealed marked pulmonary congestion and edema, whereas no overt intestinal abnormalities were observed (Figures 7F,G). Histopathological examination further demonstrated that lung lesions were characterized by extensive alveolar septal thickening and diffuse inflammatory cell infiltration. In contrast, colonic lesions were mild, with only slight lymphocyte aggregation in the villi (Figure 7H).

## 4 Discussion

Bovine coronavirus (BCoV) is an important pathogen that causes diarrhea in calves, winter dysentery in adult cattle, and respiratory symptoms. It can cause high mortality rates and serious economic losses and is associated with a risk of cross-species transmission (Nicola et al., 2008; Saif, 2010). Its harm is significant, and virus isolation faces long-term bottlenecks. As an enveloped virus, the lipid envelope of BCoV is sensitive to the external environment, and repeated freeze—thaw cycles can easily lead to loss of the fimbriae, affecting the integrity of the virus (Narula et al., 2024). Traditional separation relies on bovine primary cells or Tibetan animals, but the former are difficult to passage, and the latter is costly, making their promotion and application difficult (Zhang et al., 2018; Chen, 2025). Although multiple-passage cell lines (such as HRT-18G, HCT-8, and MDBK cells) have been used in an attempt to isolate this virus, the initial isolation efficiency is low, the cycle is long, and the culture system is unstable, which severely restricts the acquisition and in-depth research of viral resources (Lin, 2025).

The successful isolation and stable passaging of BCoV-XJHM in HRT-18G cells can be attributed to the optimized culture system developed in this study. Traditional BCoV isolation has long been hampered by the virus’s envelope fragility and low initial isolation efficiency in continuous cell lines (Narula et al., 2024; Lin, 2025). Although trypsin supplementation has been widely used to facilitate BCoV entry by cleaving the S protein (Elisabete et al., 2008; Liu et al., 2024), our study introduces two key innovations: (i) the incorporation of a trypsin pretreatment step prior to viral inoculation, and (ii) the combined optimization of trypsin B and HEPES concentrations to maintain both proteolytic activity and pH stability. This dual-factor system more effectively mimics the intestinal proteolytic environment, thereby enhancing viral adsorption and membrane fusion (Zhang and Wang, 1992). Notably, while HEPES has been used in other coronavirus culture systems (e.g., PEDV) primarily as a pH buffer (Li et al., 2017), its role in BCoV isolation has not been systematically evaluated. Our results demonstrate that optimizing HEPES concentration (100 mM in pretreatment, 50 mM in maintenance medium) in combination with trypsin B significantly improves viral replication efficiency and passage stability. This represents the first report of a trypsin-HEPES synergistic culture system for BCoV that enables continuous passaging in HRT-18G cells. Given the broad applicability of HRT-18G and similar cell lines, this optimized protocol may serve as a valuable reference for the isolation and *in vitro* propagation of other coronaviruses.

Like other RNA viruses, coronaviruses can quickly adapt to constantly changing ecological niches because of their high mutation rate and recombination frequency (Carlos et al., 1992; Patrick et al., 2009). This genetic plasticity is evident in bovine coronavirus (BCoV), which has been detected not only in cattle but also in wild and domesticated ruminants (Chasey et al., 1984; Arno et al., 2002; He et al., 2019; Decaro and Lorusso, 2020), reflecting its capacity for cross-species transmission and adaptive evolution. Given that mutations in key viral proteins are closely associated with alterations in transmissibility, tissue tropism, or host adaptation, genomic characterization of newly isolated BCoV strains is of critical importance for assessing their potential evolutionary trajectories and cross-species transmission risks. In this study, sequence analysis of the BCoV-XJHM strain revealed no common mutations in the HE, S, E, or M structural proteins; however, unique N-terminal domain mutations were identified in the relatively conserved N protein. This finding prompted a detailed comparative analysis of the structural proteins. Currently, 213 BCoV whole-genome sequences are available from NCBI. A whole-genome phylogenetic analysis of the BCoV-XJHM strain showed that it clustered into the same evolutionary branch as the prevalent strains in China. Among these strains, the BCoV-XJHM strain has the highest nucleotide similarity with the BCoV/NMG1/2022 strain and the closest genetic relationship with the GX-NN230328 strain, showing a clear trend of regional clustering evolution, which may originate from direct transmission or a common transmission chain (Pekar et al., 2025; Tandel et al., 2025). This genetic clustering is often driven by variations in key structural proteins, as the HE, S, E, and M structural proteins of coronaviruses are often subject to mutational changes that can significantly impact viral properties. For instance, mutations in the HE protein (e.g., A160S, V183F, A190V) or the S protein (e.g., H143R, A543V/S) are frequently reported hotspots associated with viral adaptation and evolution. Interestingly, the BCoV-XJHM strain identified in this study did not exhibit these commonly reported mutations in its HE and S proteins. This suggests that the presence of these specific mutations is not an absolute prerequisite for efficient viral proliferation. The functional significance of such mutations may be highly dependent on the specific viral genetic background or the host cell environment (Zhang et al., 2025; Zhao et al., 2025). In contrast to the conservation observed in other structural proteins, two notable mutations (S81A and S149A) were identified within the N protein of BCoV-XJHM. The N protein is a critical multifunctional phosphoprotein, with its N-terminal domain (NTD) playing an essential role in viral transcription and replication. Studies on the murine hepatitis virus (MHV) have demonstrated that the NTD possesses RNA helix-destabilizing activity, and mutations within this domain can be lethal by abolishing this function (Grossoehme et al., 2009; Keane et al., 2012). A comprehensive review by McBride et al further emphasizes that beyond RNA binding, the NTD is involved in viral assembly and interactions with the host cell (McBride et al., 2014). Therefore, the presence of these unique NTD mutations in BCoV-XJHM, in the absence of common HE/S mutations, suggests a potentially distinct adaptive or replicative strategy for this strain. By analogy, the S81A and S149A mutations identified in the NTD of BCoV-XJHM-N may similarly affect viral replication efficiency or host adaptation, a hypothesis warranting future investigation via reverse genetics. This study can lay a certain foundation for a deeper understanding of the sustained transmission mechanism of BCoV and the design of new prevention and control strategies.

Research shows that BCoV has the potential for cross-species infection. As an important diarrhoeal and respiratory pathogen, it mainly infects the host respiratory tract and digestive tract (Li, 2024). In a previous mouse study, oral inoculation with the BCoV HLJ-325 strain (4 × 10^8^ TCID_50_) induced significant weight loss from day 5 post-infection (*P* < 0.001) and pathological lesions including lung consolidation and colon wall thinning by day 7, despite the absence of overt clinical signs (Guo et al., 2024). Similarly, the BCoV NXWZ2310 strain caused respiratory and digestive symptoms in 7-day-old calves, with lung lobe consolidation and intestinal wall thinning observed at day 7 (Li et al., 2024).

Compared to these reported strains, the BCoV-XJHM strain isolated in this study exhibited both similarities and differences in its pathogenic profile. Similar to the HLJ-325 model, BCoV-XJHM-infected mice did not show significant diarrhea, but their weight significantly decreased from day 5 after infection (*P* < 0.05) to day 10 (*P* < 0.001), with approximately 50% of individuals achieving clinically significant weight loss. However, unlike the HLJ-325 strain that causes high viral load, severe lung consolidation, and thinning of the colon wall, BCoV-XJHM infection only leads to mild histopathological changes, mainly manifested as thickening of alveolar septa and infiltration of inflammatory cells in the lung interstitium, and only mild aggregation of lymphocytes in the microvilli of the colon, consistent with the asymptomatic phenotype reported by HLJ-325.

The low viral load of the tissue can only be detected by routine RT-PCR, indicating limited replication of this strain in non-natural host mice. It is worth noting that although oral administration is used, lung lesions appear earlier and more severe than intestinal lesions, which is consistent with the HLJ-325 model and suggests that the virus may undergo systemic spread after initial intestinal infection (Alagaili et al., 2014; Cho et al., 2024). Despite comparable inoculation doses, the pathological changes caused by BCoV XJHM are relatively mild, which may be attributed to differences in virulence, replication efficiency, or host adaptability of the strain. The induction of more severe lesions by HLJ-325 strain is associated with higher tissue viral load, while the replication limitation of BCoV XJHM may limit the degree of tissue damage. Nevertheless, the presence of low-level viruses is still sufficient to trigger inflammatory responses and pathological changes, confirming the pathogenicity of BCoV XJHM in this model (Hansa et al., 2013; Ellis, 2019; Banma et al., 2024). The early occurrence of pulmonary lesions after oral infection supports the hypothesis that the virus spreads from the intestine to distant organs (Tsunemitsu et al., 1995), and highlights the potential value of this model in studying the systemic transmission of BCoV.

However, this mouse model has inherent limitations as a surrogate host. It does not fully recapitulate the typical intestinal lesions and diarrheal symptoms characteristic of BCoV infection in cattle. Therefore, the physiological relevance of these findings needs to be further validated in the natural host (e.g., calf infection models) to better understand the mechanisms and patterns of BCoV pathogenesis and cross-species infection.

## 5 Conclusion

In this study, a stable transmissible BCoV-XJHM strain (PX693307) was successfully isolated, and the mutation characteristics and pathogenicity of its key proteins were systematically analyzed to elucidate the current genetic evolution trend of this strain and its association with potential changes in pathogenicity, providing a key theoretical basis for the prevention and control of bovine coronavirus and vaccine development.

## Data Availability

The datasets presented in this study can be found in online repositories. The names of the repository/repositories and accession number(s) can be found in the article/Supplementary material.
