# Supplementary figures and images for: Isolation, identification and pathogenicity analysis of BCoV epidemic strains in Xinjiang
Source: Front Microbiol. 2026 Mar 30;17:1788466. doi: 10.3389/fmicb.2026.1788466 (PMC13071044; doi:10.3389/fmicb.2026.1788466)

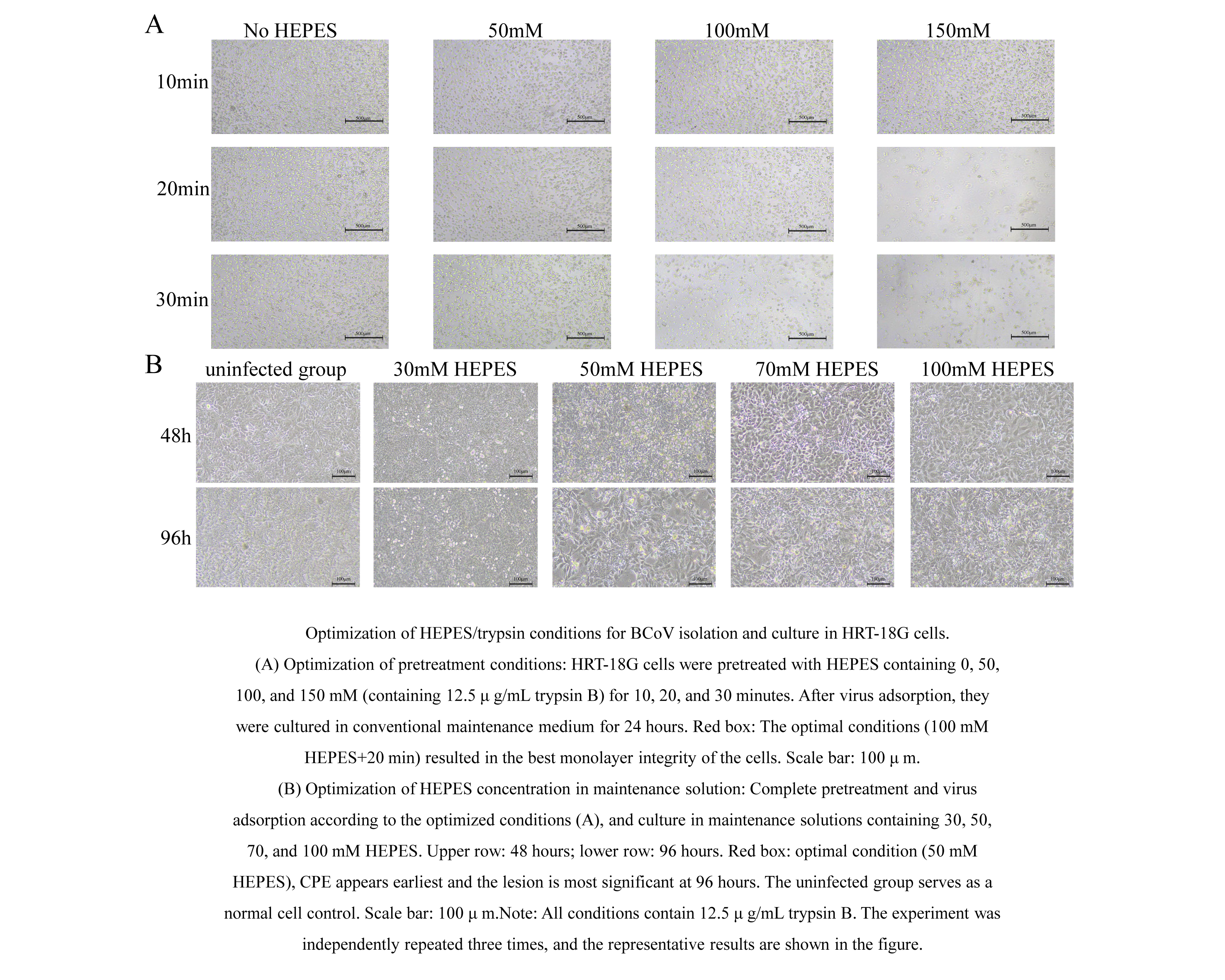

Supplement: Supplementary file 2 [file Image_1.tif]

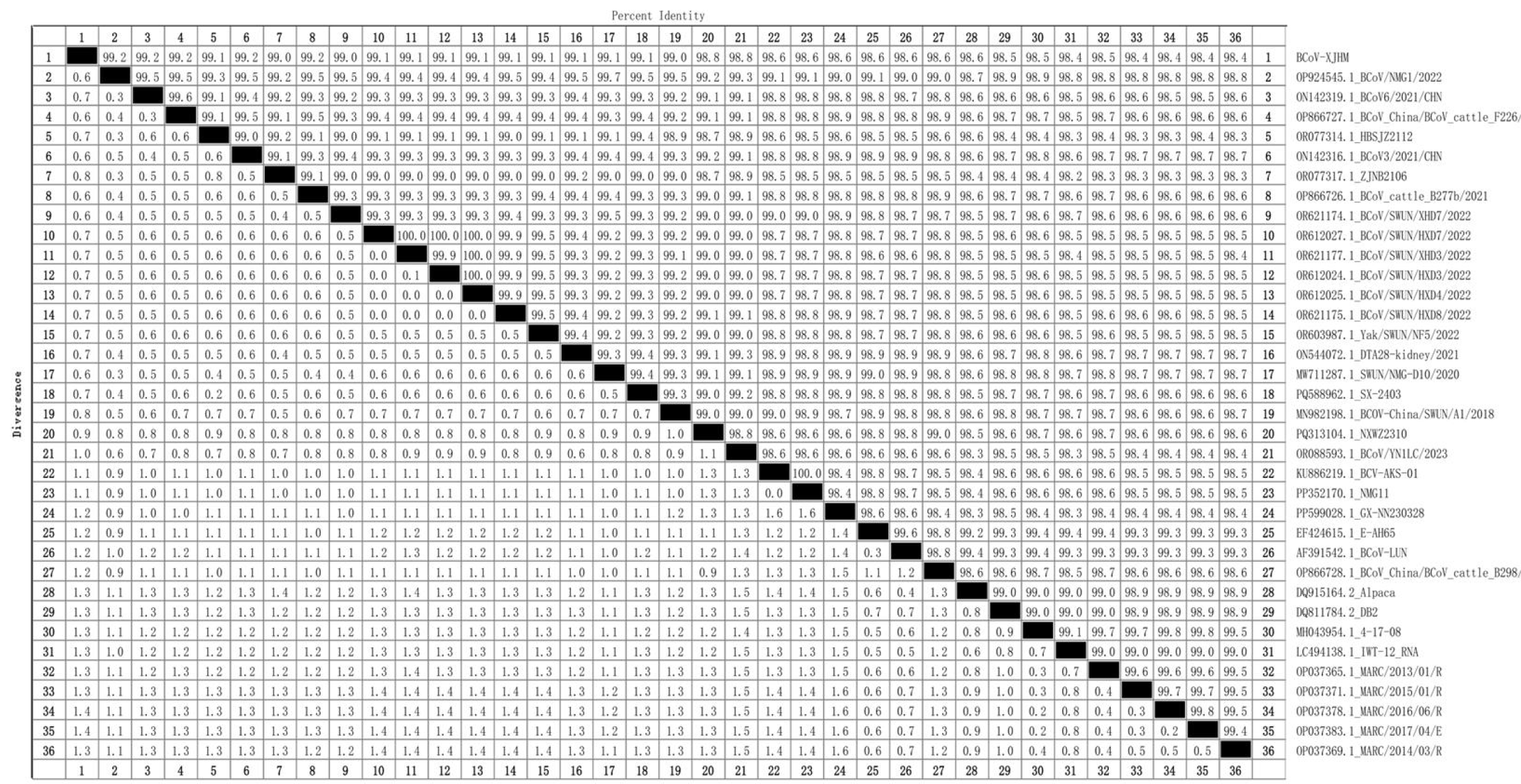

Supplement: Supplementary file 3 [file Image_2.tif]
